# Severe Anion Gap Metabolic Acidosis From Isopropyl Alcohol and Alcoholic Ketoacidosis: A Diagnostic Challenge

**DOI:** 10.7759/cureus.86071

**Published:** 2025-06-15

**Authors:** Aurora Cafuli, Devon Thorpe, Donahue Johnson, Kiana Bhola, Deepika Jain

**Affiliations:** 1 Internal Medicine, Overlook Medical Center, Summit, USA; 2 Nephrology, Overlook Medical Center, Summit, USA

**Keywords:** acetone, alcoholic ketoacidosis, anion-gap metabolic acidosis, hemodialysis (hd), isopropanol, isopropyl alcohol intoxication, osmolar gap, toxic alcohols

## Abstract

Metabolic acidosis, characterized by a decrease in blood pH due to acid accumulation or base deficit, is a life-threatening condition requiring prompt diagnosis and intervention. We describe a rare case of a 44-year-old man with severe alcohol use disorder who presented with altered mental status, acute respiratory distress, and severe wide anion gap metabolic acidosis (AGMA). Initial management with intravenous fluids, bicarbonate, and empiric fomepizole failed to improve his condition, prompting emergent hemodialysis. Volatile alcohol screening subsequently revealed elevated acetone and isopropanol levels, confirming isopropyl alcohol toxicity.

This case highlights the diagnostic challenges of volatile alcohol ingestion in AGMA and highlights the importance of considering mixed etiologies, particularly in patients with alcohol use disorder. The synergistic effects of alcoholic ketoacidosis and isopropyl alcohol toxicity led to an unusually severe presentation. Hemodialysis resulted in rapid clinical improvement, and the patient was ultimately discharged in stable condition. This case emphasizes the need for a systematic diagnostic approach, early recognition of toxic alcohol ingestion, and timely intervention in complex acid-base disturbances.

## Introduction

Metabolic acidosis, defined as a decrease in blood pH due to excess acid accumulation or loss of bicarbonate, is a common and potentially life-threatening clinical finding. A subset known as wide anion gap metabolic acidosis (AGMA) is characterized by an elevated concentration of unmeasured anions in the serum. Common etiologies of AGMA include diabetic ketoacidosis (DKA), starvation ketosis, alcoholic ketoacidosis (AKA), and ingestion of toxic alcohols such as methanol or ethylene glycol.

Isopropyl alcohol is another potentially ingested toxic alcohol, but unlike methanol and ethylene glycol, it is metabolized by alcohol dehydrogenase into acetone, a ketone that does not generate significant acidosis. As a result, isopropanol toxicity typically causes ketosis without a concurrent acidemia. However, in rare cases, particularly in patients with chronic alcohol use disorder, isopropanol ingestion may occur alongside AKA or organ dysfunction, producing complex mixed acid-base disturbances that obscure diagnosis.

This case highlights a rare but clinically significant presentation of AGMA caused by the combined effects of AKA and isopropyl alcohol ingestion. The patient's severe acidosis and markedly elevated osmolar gap initially raised concern for methanol or ethylene glycol ingestion. Ultimately, early clinical suspicion, broad toxicologic screening, and timely initiation of hemodialysis were crucial in identifying and managing this mixed toxic-metabolic presentation. This report emphasizes the importance of maintaining a broad differential and recognizing coexisting metabolic derangements in patients with alcohol use disorder presenting with severe acidosis.

## Case presentation

A 44-year-old man with a history of alcohol use disorder, depression, and anxiety presented to the emergency department in acute respiratory distress. He was disoriented, had difficulty ambulating, and had non-bloody, non-bilious emesis. He was discovered by his family in an unresponsive state, with multiple empty alcohol bottles nearby. The patient denied fever, headache, or recent use of over-the-counter medications or herbal remedies. 

Upon arrival, he was hypotensive (BP 86/50 mmHg), tachycardic (HR 109 bpm), tachypneic (27 breaths/min), and hypothermic (temperature 95.9°F) with a BMI of 25.8 kg/m^2^. Physical examination revealed a severely ill-appearing patient with a fruity odor to his breath, abdominal tenderness with guarding (without rebound tenderness), and decreased bowel sounds.

Given his history of chronic alcohol use disorder, an initial differential included acute ethanol ingestion, AKA, DKA, uremia, and ingestion of toxic alcohols such as methanol, ethylene glycol, or salicylates. The absence of hyperglycemia and a normal blood glucose level effectively ruled out DKA as the cause.

Serum bicarbonate level of 8 mmol/L prompted arterial blood gas (ABG) analysis, which revealed a pH of 7.16, bicarbonate 5.9 mEq/L, and PaCO₂ 17 mmHg, consistent with severe wide AGMA. Delta-delta analysis was performed to assess for a mixed acid-base disorder. The change in anion gap (44−12=32) compared to the change in bicarbonate (24−5.9=18.1) yielded a delta ratio of approximately 1.8, suggesting a high anion gap acidosis with concurrent metabolic alkalosis or chronic respiratory alkalosis. Winter's formula (PaCO₂=1.5×HCO₃⁻+8±2) predicted a PaCO₂ of approximately 17 mmHg, confirming appropriate respiratory compensation.

Other significant laboratory studies showed elevated serum creatinine of 3.66 mg/dL (baseline unknown), blood urea nitrogen (BUN) of 30 mg/dL, anion gap of 44, beta-hydroxybutyrate (BHB) of >45, lactic acid of 0.7, measured serum osmolality of 326 mOsm/kg, calculated osmolality of 388 mOsm/kg, and osmolar gap of 37.1 mOsm/kg. The osmolar gap, calculated as the difference between measured and calculated osmolality, is a useful diagnostic clue when it is >10 mOsm/kg, indicating the presence of unmeasured osmotically active solutes such as methanol, ethylene glycol, or isopropanol. However, it is not specific and must be interpreted in the clinical context. Table 1 summarizes the laboratory findings on admission and discharge. 

**Table 1:** Admission and discharge laboratory values BUN: blood urea nitrogen; AST: aspartate aminotransferase; ALT: alanine aminotransferase; ALP: alkaline phosphatase

Lab test	On admission	On discharge	Reference range
Glucose	88 mg/dL	100 mg/dL	70-100 mg/dL
BUN	30 mg/dL	7 mg/dL	7-18 mg/dL
Creatinine	3.66 mg/dL	0.89 mg/dL	0-1.3 mg/dL
Sodium	133 mmol/L	140 mmol/L	135-145 mmol/L
Potassium	4.4 mmol/L	3 mmol/L	3.2-4.9 mmol/L
Chloride	81 mmol/L	108 mmol/L	95-110 mmol/L
AST	145 U/L	115 U/L	15-37 U/L
ALT	76 U/L	52 U/L	12-59 U/L
ALP	91 U/L	257 U/L	45-117 U/L
CO2	8 mmol/L	26 mmol/L	21-32 mmol/L
Anion gap	44 mmol/L	6 mmol/L	5-50 mmol/L
Calcium	9 mg/dL	8.8 mg/dL	8.5-10.1 mg/dL
Phosphorus	10.8 mg/dL	2.2 mg/dL	2.5-4.8 mg/dL
Osmolality	326 mOsm/kg	Not repeated	275-300 mOsm/kg
Beta-hydroxybutyrate	>46 mg/dL	Not repeated	0.2-2.8 mg/dL
Lactic acid	0.7 mmol/L	Not repeated	0.4-2 mmol/L

Despite the suspicion of AKA, the presence of high BHB levels, normal glucose, and absence of lactic acidosis suggested a mixed or alternative etiology. Ethanol levels were mildly elevated at 27 mg/dL, insufficient to account for the severe AGMA. The anion gap was likely multifactorial, contributed to by uremia, elevated BHB, and ethanol. Concern grew for toxic alcohol ingestion [1]. 

Due to the absence of local testing capabilities for volatile alcohols, urine and serum specimens were submitted to an out-of-state reference laboratory for analysis. Empiric therapy was initiated expeditiously based on clinical judgment, including bicarbonate infusion, correction of electrolyte abnormalities, administration of fomepizole, and subsequent initiation of hemodialysis under consensus guidelines for toxic alcohol ingestion. Although fomepizole is not routinely indicated in isopropanol toxicity, it was initiated empirically due to diagnostic uncertainty and concern for possible methanol or ethylene glycol ingestion pending confirmatory results [2].

Volatile screen later confirmed elevated levels of acetone (62 mg/dL) and isopropanol (21 mg/dL), confirming the exposure to isopropyl alcohol. Serum and urine tests for methanol and ethylene glycol were negative, ruling out the presence of these substances. The absence of visual symptoms, lack of calcium oxalate crystalluria, and no retinal findings also made methanol and ethylene glycol ingestion less likely [2]. Table 2 summarizes the relevant toxicology and metabolic studies. 

**Table 2:** Toxicology and metabolic studies

Test	Result	Reference range
Osmolar gap	37.1 mOsm/kg	<10 mOsm/kg
Salicylates	<2.8 mg/dL	2.8-20 mg/dL
Ethanol	27 mg/dL	<10 mg/dL
Ethylene glycol	<20 mg/dL	<20 mg/dL
Methanol (methyl alcohol)	<0.010	<0.010
Methanol (urine)	Not detected	N/A
Ethanol (urine)	11 mg/dL	<10 mg/dL
Acetone (urine)	62 mg/dL	<10 mg/dL
Isopropanol (urine)	21 mg/dL	<10 mg/dL

The patient was started on naltrexone for alcohol use disorder, but this was discontinued due to mild transaminitis. He was ultimately discharged with plans to follow up with Alcoholics Anonymous (AA) and begin a chemical dependence intensive outpatient program (CD-IOP) with close follow-up with his primary care provider. 

## Discussion

This case presents a rare and complex presentation of wide AGMA resulting from the combined effects of isopropyl alcohol toxicity and chronic AKA. Isopropyl alcohol is primarily metabolized in the liver to acetone, a ketone that is not acidogenic but may worsen underlying ketosis. This patient's presentation illustrates how concurrent isopropanol ingestion and chronic alcohol use can potentiate severe metabolic derangements.

AKA is a well-recognized consequence of prolonged ethanol use, particularly in malnourished individuals with glycogen depletion. In AKA, the shift from glucose to fatty acid metabolism results in the increased production of ketone bodies, particularly BHB [2,3]. Markedly elevated BHB levels (>45 mg/dL in our patient) reflect substantial metabolic stress and are a hallmark of AKA, illustrating an important metabolic adaptation to chronic ethanol exposure [3]. However, the absence of hyperglycemia, normal blood glucose, and normal lactate levels helped differentiate this presentation from DKA [4].

Isopropanol, unlike methanol or ethylene glycol, exerts direct central nervous system depressant effects and is metabolized by hepatic alcohol dehydrogenase to acetone. While acetone itself is not acidogenic, accumulation in the context of ketosis, along with renal and hepatic dysfunction, may exacerbate acid-base disturbances. Although no clinical studies have directly quantified impaired acetone clearance in patients with chronic liver or kidney disease, it is physiologically plausible that hepatic dysfunction, particularly in the setting of chronic alcohol use, could reduce acetone metabolism, as hepatic mitochondrial oxidation is its primary clearance pathway. Furthermore, ethanol competitively inhibits alcohol dehydrogenase, potentially delaying isopropanol metabolism and prolonging toxicity [2,5,6]. 

This case also highlights the diagnostic challenges of identifying mixed alcohol ingestions. Toxic alcohol exposure may be obscured in individuals with alcohol use disorder due to overlapping metabolic abnormalities. While methanol and ethylene glycol poisoning were initially suspected due to the wide AGMA, toxicology screens for these substances were negative. The patient's ethanol level (27 mg/dL) was insufficient to explain the severity of acidosis. Definitive diagnosis was made via volatile alcohol screening, which confirmed elevated acetone and isopropanol levels [1].

To reduce diagnostic delays in similar cases, protocols should include bedside osmolar gap calculations and point-of-care ketone testing. In this case, the osmolar gap was significantly elevated at 37.1 mOsm/kg. While an osmolar gap of >10 mOsm/kg suggests unmeasured solutes such as toxic alcohols, it is nonspecific and may also occur in AKA. These findings must be interpreted within the broader clinical context of ingestion history, lab values, and mental status [1,7-9].

Although hemodialysis is not typically required in isopropyl alcohol toxicity, the combination of severe AGMA, clinical deterioration, and elevated volatile levels warranted dialysis in this case [2,10,11]. Hemodialysis facilitated the rapid removal of both isopropanol and acetone, resulting in the reversal of central nervous system depression and correction of acid-base disturbances. Notably, the patient's hemodynamic stability and mental status improved significantly within 24 hours of initiating treatment.

In the setting of renal and hepatic dysfunction, the accumulation of isopropanol and acetone may contribute to metabolic instability. In severe isopropanol toxicity, dialysis is warranted due to the dialyzable nature of these compounds [2,11]. Once stabilized, the patient was initiated on naltrexone for alcohol use disorder; however, this was discontinued due to mild transaminitis, emphasizing the importance of close hepatic monitoring when initiating pharmacotherapy in patients with underlying liver dysfunction [12].

This case emphasizes the importance of maintaining a broad differential when evaluating wide AGMA, particularly in patients with chronic alcohol use disorder. Early recognition of toxic alcohol ingestion, combined with targeted diagnostics such as osmolar gap analysis and volatile screening, can help identify uncommon or mixed metabolic etiologies. Timely intervention, including hemodialysis when appropriate, and close collaboration with toxicology experts are essential in improving patient outcomes in complex poisoning cases.

## Conclusions

This case highlights an atypically severe presentation of wide AGMA in a patient with chronic alcohol use disorder, driven by the synergistic effects of AKA and isopropyl alcohol ingestion. While isopropyl alcohol is not typically acidogenic, its accumulation, when combined with elevated BHB, uremia, and impaired hepatic and renal clearance, contributed to a profound metabolic disturbance. Volatile alcohol screening, along with the use of diagnostic tools such as Winter's formula and delta-delta calculations, was essential in identifying the underlying cause after more common toxins were excluded. Early recognition of a mixed metabolic etiology and timely initiation of hemodialysis were critical in reversing the patient's clinical decline. This case reinforces the importance of early recognition, targeted diagnostics, and toxicology consultation when managing complex acid-base disorders in patients with alcohol use disorder.
